# Pioglitazone Attenuates Cystic Burden in the PCK Rodent Model of Polycystic Kidney Disease

**DOI:** 10.1155/2010/274376

**Published:** 2010-11-01

**Authors:** Bonnie L. Blazer-Yost, Julie Haydon, Tracy Eggleston-Gulyas, Jey-Hsin Chen, Xiaofang Wang, Vincent Gattone, Vicente E. Torres

**Affiliations:** ^1^Department of Biology, Indiana University Purdue University at Indianapolis, 723 West Michigan Street, Indianapolis, IN 46202, USA; ^2^Department of Anatomy and Cell Biology, Indiana University School of Medicine, Indianapolis, IN 46202, USA; ^3^Department of Pathology and Laboratory Medicine, Indiana University School of Medicine, Indianapolis, IN 46202, USA; ^4^Division of Nephrology and Hypertension, Mayo Clinic, Rochester, MN 55905, USA

## Abstract

Polycystic kidney disease (PKD) is a genetic disorder characterized by growth of fluid-filled cysts predominately in kidney tubules and liver bile ducts. Currently, the clinical management of PKD is limited to cyst aspiration, surgical resection or organ transplantation. Based on an observation that PPAR*γ* agonists such as pioglitazone and rosiglitazone decrease mRNA levels of a Cl^−^ transport protein, CFTR (cystic fibrosis transmembrane conductance regulator), and the Cl^−^ secretory response to vasopressin in cultured renal cells, it is hypothesized that PPAR*γ* agonists will inhibit cyst growth. The current studies show that a 7- or 14-week pioglitazone feeding regimen inhibits renal and hepatic bile duct cyst growth in the PCK rat, a rodent model orthologous to human PKD. These studies provide proof of concept for the mechanism of action of the PPAR*γ* agonists and suggest that this class of drugs may be effective in controlling both renal and hepatic cyst growth and fibrosis in PKD.

## 1. Introduction

Agonists of the peroxisome proliferator activator receptor gamma (PPAR*γ*) have pleiotropic effects on intermediary metabolism. Two of these agents, rosiglitazone and pioglitazone, are approved for clinical use as insulin-sensitizing agents in the treatment of type II diabetes. One of the major side effects of PPAR*γ* treatment is fluid retention that can, in some instances, result in overt edema [1–3]. A renal collecting duct-specific knockout of PPAR*γ* in rodent models abrogates the drug-induced fluid retention, suggesting that the effect arises from alterations in electrolyte and/or fluid transport in the distal nephron [4, 5]. Studies in a cell culture model of the principal cell type of the distal nephron have demonstrated that PPAR*γ* agonists inhibit cAMP-stimulated anion transport and the mRNA expression of the cystic fibrosis transmembrane conductance regulator (CFTR), a Cl^−^ channel found in the apical membrane of this cell type [6]. 

Polycystic kidney disease (PKD) is a genetic disease with both autosomal dominant (ADPKD) and autosomal recessive (ARPKD) forms [7, 8]. ADPKD is the more prevalent form, striking approximately 1 in 1000 in the adult population and presenting as slow growing, fluid-filled cysts which form predominately in the kidney. Progressive cyst growth and fibrosis in the surrounding tissue generally compromise organ function after middle age and lead to end-stage renal disease after the fifth decade. ARPKD affects children in the neonatal period and is characterized by tubular dilation. Both forms of PKD have liver involvement with cysts that arise from cholangiocytes, an epithelial cell type lining the hepatic bile ducts. 

The genes that are mutated in PKD encode proteins found in the primary cilia, cytoplasmic vesicles, endoplasmic reticulum, and cell-cell and cell-extracellular matrix contacts. These proteins are either transient receptor potential Ca^2+^ channels or proteins that regulate this class of Ca^2+^ channels [7, 8]. Disease-associated decreases in intracellular Ca^2+^ concentrations modulate intracellular signaling pathways including those regulating cAMP and Cl^−^ channels. 

It is widely accepted that secretion of ions and fluid by the cyst-lining epithelial cells contribute to cyst expansion. Studies performed over a decade ago indicate that renal cyst formation in PKD is driven by anion (Cl^−^ or HCO_3_^−^) secretion [9–11], and more recent studies have shown a remarkably similar profile in freshly isolated bile duct epithelia [12]. Inhibitor studies and electrophysiological analyses have shown that CFTR is the Cl^−^ channel responsible for secretion in both kidney and biliary cysts [10, 12]. Based on the previous finding that PPAR*γ* agonists inhibit CFTR expression [6], the current studies were designed to test the efficacy of a PPAR*γ* agonist, pioglitazone, in inhibiting cyst growth. 

Of the several rodent models for PKD, we chose to use the PCK rat model because the genetic mutation in this animal is orthologous to human ARPKD, while the animals also express many of the phenotypic characteristics of human ADPKD [13, 14]. The animals develop both kidney and liver fibrocystic diseases and live long enough to facilitate long-term treatment protocols. This model was used in preclinical testing of both the renal V2 receptor antagonist and the somatostatin agonist as treatment options for PKD [15–17].

## 2. Subjects and Methods

### 2.1. Animals

PCK rats were purchased from Charles River Laboratories, Inc. (Wilmington, MA) or were bred in the colonies at Indiana University School of Medicine (IUSM) or the Mayo Clinic. Institutional Animal Care and Use Committees at each institution approved all protocol procedures. The amount of pioglitazone added to the chow was based on the estimated animal consumption of the base diets.

### 2.2. Study Design and Protocol

#### 2.2.1. IUPUI

After weaning, at four weeks of age, rodents were randomly separated into treatment and control groups. The treatment group was fed Purina no. 5002 Labdiet supplemented with pioglitazone calculated to provide 20 mg/kg BW when provided *ad libitum*. Control rodents were fed unsupplemented Purina no. 5002 Labdiet. At week 18, rodents were anesthetized with 100 mg/kg intraperitoneally injected sodium pentobarbital. Blood was collected via intracardiac puncture and analyzed for plasma chemistries at the Clarion Pathology Laboratories of the Indiana University School of Medicine. After a laparotomy, the organs were flushed with saline, and the left kidney and right liver lobe were collected and frozen in liquid nitrogen. The remaining kidney and liver were infused with 4% paraformaldehyde, removed, and stored in 4% paraformaldehyde for histology. Kidney and liver were embedded in paraffin and sectioned transversely (4 *μ*M sections).

#### 2.2.2. Mayo

Treatment with pioglitazone was started at the time of weaning or three weeks of age. Pioglitazone (Takeda Chemical Industries) was added to the food at concentrations estimated to provide 4 or 20 mg/kg/day. The control animals received unsupplemented diet. At the end of each study, rats were anesthetized with ketamine 60 mg/kg and xylazine 10 mg/kg, IP. Blood was obtained by cardiac puncture for determination of plasma creatinine, BUN, electrolytes, bilirubin, liver enzymes, and glucose. The right kidney, part of the liver, the pancreas, and the spleen were placed into preweighed vials containing 10% formaldehyde in phosphate buffer (pH 7.4). These tissues were embedded in paraffin for histological studies. The left kidney and part of the liver were immediately frozen in liquid nitrogen.

### 2.3. Histological Analyses

#### 2.3.1. IUPUI

 Medial transverse kidney sections were stained with hematoxylin, and eosin and cyst volumes were analyzed using point count stereology methods on deidentified sections [18]. Kidney and liver fibrosis was assessed with picrosirius red staining. Kidney fibrosis was graded on a scale of 1–4 (1-normal, 2-slight, 3-moderate, and 4-severe) in both the cortex and the juxtamedullary areas of the kidney. Similarly, liver fibrosis was assessed on a scale of 1–4 (1-less than 1% fibrosis, 2-less than 5% fibrosis, 3-less than 20% fibrosis, and 4-greater than 20% fibrosis). All scoring was done by trained scientists blinded to the identity of the sections.

#### 2.3.2. Mayo

Four *μ*m transverse tissue sections of the kidney, including cortex, medulla, and papilla, and of the liver were stained with hematoxylin-eosin and picrosirius red. Whole transverse tissue sections stained with hematoxylin-eosin were used to measure cyst volumes. Renal and hepatic fibrosis was scored using the picrosirius red staining. Image analysis procedures were performed without knowledge of group assignments using Meta-Morph software (Universal Imaging, West Chester, PA). Stained sections were visualized and digital images were acquired using a high-resolution Nikon Digital camera and displayed on the monitor. Definition of interested structures and exclusion of fields too damaged to be analyzed were achieved by interactively applied techniques of enhancement. Colored thresholds were applied at levels which separate cysts from noncystic tissue. Kidney and liver fibrosis were graded as above.

### 2.4. Electron Microscopy Studies

Small pieces of the perfusion fixed kidney were processed for electron microscopic immunogold labeling in the IU School of Medicine Electron Microscopy Center (http://www.anatomy.iupui.edu/corefac/emcenter.php) using a standard postembedding labeling technique. Kidney pieces which had visible cysts were trimmed to 2 × 2 mm segments which were dehydrated and embedded in Unicryl (Electron Microscopy Sciences, Hatfield, PA). Thin sections (70–90 nm) were mounted on Formvar/carbon coated nickel grids, rinsed with PBS, and grids placed in blocking buffer (2% BSA, 0.1% Cold Water Fish Gelatin, and 0.1% Tween in PBS) for 30–45 minutes. The grids were then placed in the primary antibody (mouse monoclonal anti-CFTR antibody, 596, 1 : 5, kindly provided by John R. Riordan, University of North Carolina) overnight at 4°C in a humid chamber. The grids were then rinsed with PBS and floated on drops of the appropriate secondary antibody with attached 10 nm colloidal gold (AURION, Hatfield, PA) for 2 hours at room temperature and rinsed with PBS. To more firmly adfix the antibody bindings, grids were placed in 2.5% Glutaraldehyde in 0.1 M Phosphate buffer for 15 minutes. After rinsing in distilled water, the grids were stained for contrast with uranyl acetate. The samples were viewed with a Tecnai Bio Twin transmission electron microscope (FEI, Hillsboro, OR). The primary and secondary antibodies were diluted in an incubation buffer containing 0.1% BSA-c (AURION), 0.05% Tween in PBS.

### 2.5. Statistical Analysis

Comparisons between treatment and gender groups were performed using the Student's *t*-test for unpaired samples. Two-tailed *P*-values (*P* < .05) were used to denote statistical significance.

## 3. Results

The data presented below are the combination of studies performed independently in two institutions. The 7-week feeding study was conducted at the Mayo Clinic while the 14-week study was performed at Indiana University—Purdue University Indianapolis (IUPUI). The results of the independent studies were combined after completion of each series of experiments. Slight differences in experimental protocols and measured parameters are a function of the independence of study design between the institutions.

**Figure 1:**
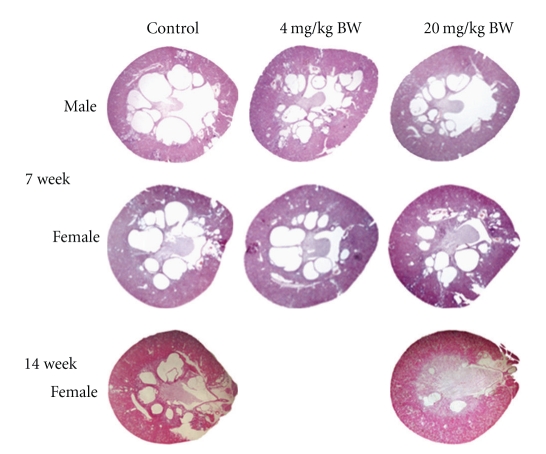
Effect of Pioglitazone on renal cysts in PCK rat model. The images show transverse sections of kidneys from animals that had been fed for 7 or 14 weeks with normal chow (control) or chow supplemented with pioglitazone to approximate a daily treatment of 4 or 20 mg/kg BW as indicated on the figure.

PCK rats were fed either a control diet or a diet supplemented with pioglitazone starting after weaning. Both male and female animals were used in the 7-week study while the 14-week study was conducted in female rats. The data are analyzed in a gender-specific manner. The administration of pioglitazone at a dose of 20 mg/kg body weight was accompanied by a significant decrease in renal cyst burden in the male animals after only 7 weeks of pioglitazone treatment (Table 1; Figure 1). While the decrease in renal cyst burden in female rats did not reach statistical significance after 7 weeks of feeding (*P* = .06), there was a significant decrease in the pioglitazone-fed females after 14 weeks (Table 1; Figure 1). Pioglitazone was also effective in decreasing the hepatic cyst burden after 7 or 14 weeks in female rats, but had no detectable effect on the milder liver cystic disease observed in the male rats after 7 weeks. 

Since PPAR*γ* agonists exhibit dose-dependent, pleomorphic effects, we also determined the efficacy of a lower dose of pioglitazone (4 mg/kg BW) on the development of cystic disease. Interestingly, this dose was as effective as the higher dose in reducing the renal cyst burden in both female and male animals (Table 2). The lower dose also decreased liver weight as a percentage of body weight in both male and female animals.

There was a trend for pioglitazone to reduce renal and hepatic fibrosis. Possibly because fibrosis is mild at early stages of the disease in this animal model, the antifibrotic effect of pioglitazone was not consistently observed and reached statistically significance in only certain groups. In the seven-week study, pioglitazone was effective on renal disease in males on the low dose and on liver disease in females on the high dose, while in the fourteen week study the agonist was effect on renal cortical but not medullary fibrosis in the female rats (Tables 1 and 2).

Table 3 shows the results of serum analyses conducted on the female animals that were fed for 14 weeks. No changes in serum parameters were found except an increase in serum albumin. A similar profile was obtained at the 7th week time point (data not shown).

**Figure 2:**
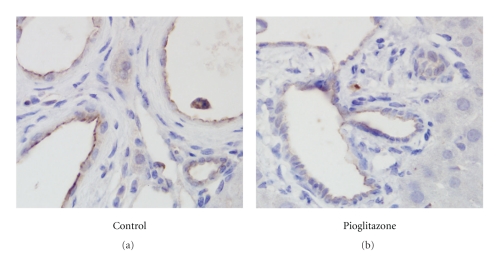
Immunostaining for CFTR in the liver of control and pioglitazone-treated PCK rats after 14 weeks of drug treatment. Control (a) or 20 mg/kg BW pioglitazone-treated (b) liver sections were immunostained for CFTR using mAb 596. Shown in both panels is the CFTR staining (brown) in the apical membranes of cyst-lining biliary epithelial cells. 1000x magnification.

To elucidate a mechanism for the action of pioglitazone in cyst formation, the apical membrane expression of CFTR in epithelial cells surrounding cysts was examined using the monoclonal antibody 596 [19]. Figure 2 illustrates CFTR immunoreactivity in cholangiocytes lining the liver cysts. The CFTR-positive apical membranous staining seen in the control tissue was diminished in the pioglitazone-treated animals. Insufficient CFTR was present in the renal slices for unambiguous detection of changes at the light level although the CFTR channel is clearly present based on functional studies [6, 9–11]. 

**Figure 3:**
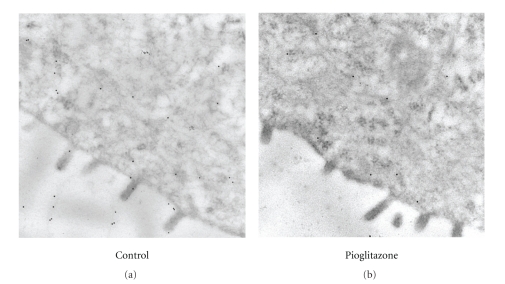
Immunostaining for CFTR in the liver of control and pioglitazone-treated PCK rats after 14 weeks of drug treatment. Control (a) or 20 mg/kg BW pioglitazone-treated (b) liver sections were immunostained for CFTR with mAb 596 CFTR antibody followed by a gold-labeled secondary antibody. Shown in both panels is the CFTR staining (spherical dots) in the apical membrane sections of epithelial cells surrounding the bile duct cysts. 49 000x magnification.

To provide a better quantitative approach, gold-labeled immunoelectron microscopy was used to assess the density of CFTR expression at the apical plasma membrane of the bile duct cysts (Figure 3). Sections from 7 drug-treated and 6 control rats such as the ones shown in figure 3 were subjected to analysis by counting the number of gold particles bound to the surface of the apical membrane and expressing this quantity as the number of particles per linear *μ*m of apical surface. A total of 36.24 linear *μ*m of apical surface was examined in the treated animals and 35.08 *μ*m was examined from the control animals. The pioglitazone-treated tissue showed a statistically significant decrease (*P* = .009) in the number of apically localized immunolabeled CFTR antibody particles. The biliary cysts showed an average of 1 gold-labeled particle per 0.23 linear *μ*m of apical surface in the animals on the control diet and an average of 1 per 6.04 *μ*m in the animals treated with pioglitazone.

An overall decrease in CFTR labeling was also observed in the immuno-gold labeled EM of renal cyst sections taken from animals treated with pioglitazone for 14 weeks. However, the lower abundance of CFTR expression in the renal cysts from both the treated and untreated animals precluded a statistical assessment for an alteration of CFTR immunoreactivity in the apical membrane.

## 4. Discussion

A variety of full and partial PPAR*γ* agonists inhibit vasopressin-stimulated Cl^−^ secretion via CFTR in the MDCK-C7 (Madin Darby Canine Kidney, clone 7) cell culture model of the principal cells lining the distal nephron. The dose response curves for agonist inhibition of transepithelial Cl^−^ transport paralleled the EC_50_ for receptor transactivation with a log unit leftward shift, suggesting that the effect on Cl^−^ secretion is very sensitive and manifested within serum concentrations at or below those used to treat insulin sensitivity [6]. Based on these data, it was hypothesized that a PPAR*γ*-mediated decrease in the number of CFTR channels in the epithelial cells that line PKD kidney and liver cysts would have the effect of decreasing Cl^−^ secretion into the cyst lumen, thereby decreasing the osmotic driving force for fluid accumulation. 

Previous studies have examined PPAR*γ* agonist effects on PKD in rodent models. A functional knockout of PKD1, the gene encoding the polycystin 1 protein, resulted in an embryonic lethal phenotype in mice presenting with hydrops, cardiac conotruncal defects, and renal cystogenesis [20]. The embryos show decreased cardiac levels of c-MYC, and abnormal metabolism of E-cadherin and PECAM-1 in renal tubules, and hemorrhagic diathesis, the most serious of which is hemorrhagic pericardial effusions. Maternally administered pioglitazone at high doses (80 mg/kg/day) improved survival of the embryos by several days, ameliorated the cardiac defect and decreased the degree of renal cystogenesis by an unknown mechanism [20]. In a similar study based on the antineoplastic properties of pioglitazone, the authors evaluated the drug's effect in PC-Pkd1-KO mice, a rapidly progressing model of ADPKD [21]. Feeding the nursing mothers, and then the pups, did not alter renal function, cell proliferation, apoptosis, or cyst formation. However, the PPAR*γ* agonist did increase survival and had an antihypertensive effect. Dai and colleagues used the more slowly progressing, Han:SPRD rat model to show that rosiglitazone (10 mg/kg BW/day) was able to delay the progression of kidney cysts and preserve renal function [22]. The authors suggested that the mechanism of action may be due to PPAR*γ* agonist downregulation of an abnormally activated *β*-catenin signaling pathway as well as the anti-inflammatory and antifibrotic effects of this class of drugs. Cyst formation in this nonorthologous model may be difficult to correlate with human disease because the renal cysts are primarily proximal in origin and the model does not demonstrate the bile duct cyst formation that is common in human PKD.

Our studies, using an orthologous, slowly progressing rat model, address the effects of PPAR*γ* agonist treatment early in the course of the disease but after the embryonic or neonatal period. This preclinical model more closely mimics the situation in potential human treatment both in the timing of when the agonists would be administered and in the nature of the slow disease progression. The data represent two independent studies that were combined retrospectively. Although there are differences in experimental protocols, particularly with regard to the length of the feeding duration, the strength of the findings lies in the consistency of the final results.

In the PCK model as little as 7 weeks of pioglitazone reduced liver cyst burden in the female animals and renal cyst burden in the male animals. After 14 weeks on a pioglitazone-supplemented diet, there were significant decreases in both kidney and liver in both sexes. Cross-sectional images of the kidney from littermates on control or pioglitazone-supplemented diets for 14 weeks indicate that the decrease in cyst burden is predominately due to a decrease in cyst size. The “decrease” in cyst size is likely due to a slower growth of the cysts in the presence of pioglitazone.

Similar to human ADPKD, the PCK rat exhibits some gender dimorphism in the severity of the renal and hepatic pathology [23]. Up to 18 weeks, the pathological alterations are relatively similar in both males and females. Starting at about 18 weeks of age, male PCK rats accelerate their renal cystic enlargement resulting in a more severe renal dysfunction compared to the females [13, 23]. However, female PCK rats develop a greater liver cyst volume density starting at 18 weeks [23]. The gender differences in efficacy during early pioglitazone treatment may be due to subtle, earlier gender differences in disease progression.

The serum profiles after 14 weeks of feeding indicate that kidney function in these animals is relatively well preserved despite the cystic burden. The blood urea nitrogen is within the normal range and is not significantly altered by treatment with pioglitazone. While bilirubin levels are in the normal range, liver function as assessed by liver enzymes shows a compromised hepatic function and this is substantiated by the low levels of total protein and albumin in the serum. Pioglitazone treatment did not correct the high levels of liver enzymes but did increase the serum albumin concentration. 

The effect of pioglitazone on tissue fibrosis is more difficult to assess. In other diseases, the use of PPAR*γ* agonists has been correlated with decreases in inflammatory responses and tissue fibrosis, two related processes that contribute to organ function decline in PKD. Recent studies have shown that PPAR*γ* agonists can reduce fibrosis in tissues as diverse as epidermis [24], lung [25, 26], kidney [27–30], and liver [31–34]. These agents are effective in multiple fibrotic diseases suggesting a commonality in the drug targets. Since fibrosis is mild in early stages of disease progression in this model of PKD, it is not surprising that our results regarding the effect of PPAR*γ* agonists are somewhat inconsistent. Longer term studies will be necessary to clarify potential effects on fibrotic development.

The importance of CFTR activity in renal and bile duct cyst formation is based on functional studies. Due to low abundance of the protein, few studies have succeeded in detecting the protein in the apical membrane of native renal cysts by immunohistochemical means. The demonstration that PPAR*γ* agonists diminish CFTR levels was initially established in cultured cells [6]. The current studies were able to extend the previous work to native tissue and demonstrate that in biliary cysts, pioglitazone treatment results in a statistically significant lower amount of the CFTR protein in the apical membrane of the cholangiocytes lining the cysts. These studies extend the cultured cell experiments and provide the proof-of-principle for a mechanism whereby the PPAR*γ* agonists inhibit the progression of cyst growth.

The idea to use CFTR inhibitors to treat PKD is not new. Several groups have proposed this approach [35, 36]. The recent discovery that PPAR*γ* agonists inhibit CFTR synthesis provides additional possibilities using drugs that have already undergone long-term testing in humans. Although the PPAR*γ* agonists can have side effects, such as fluid retention and weight gain via adipogenesis, these are relatively mild. However, strategies that minimize adverse events are very important when considering treatments for a chronic disease such as PKD. Since renal cell culture studies indicate that agonist effect on CFTR occurs at concentrations approximately an order of magnitude lower than those necessary to stabilize insulin action [6], lower drug doses may still be effective with minimal side effects. Of note, the lower dose of pioglitazone was as effective in our study as the higher dose. 

PPAR*γ* agonists alter the density of CFTR expression in the plasma membrane but do not interfere with the action of vasopressin on water transport or cause polyuria as observed with vasopressin V2 receptor antagonists. Moreover, PPAR*γ* agonists appear to be effective not only in the kidney but in other tissues where cyst formation and growth are dependent on CFTR-driven ion and fluid secretion. Therefore, their possible value, either alone or in combination with other promising therapies, in the treatment of PKD deserves further consideration.

## 5. Conclusions

The studies show that a 7- or 14-week pioglitazone feeding regimen inhibits renal and hepatic bile duct cyst growth in the PCK rat, a rodent model orthologous to human PKD. In bile duct cysts, the effects were accompanied by a decrease in the apical expression of CFTR, indicating that the mechanism of action *in vivo* matches previous cell culture data that show a PPAR*γ*-mediated decrease in CFTR expression. In addition, in some studies, the degree of fibrosis was diminished in pioglitazone-treated animals. These studies provide proof-of-concept for the mechanism of action of the PPAR*γ* agonists and suggest that these drugs may be effective in controlling both renal and hepatic cyst growth and fibrosis in PKD.

## Figures and Tables

**Table 1:** Effect of pioglitazone (20 mg/kg body weight) on fibrocystic disease in the PCK rat model.

7-week feeding protocol	Female animals				Male animals			
	Control *n* = 8	Pioglitazone *n* = 6	*P*-value and significance		Control *n* = 5	Pioglitazone *n* = 8	*P*-value and significance	
Body weight (gr)	239 ± 9.48	224 ± 5.04	.21	NS	347 ± 9.50	328 ± 9.45	.20	NS
Kidney weight (gr)	3.74 ± 0.22	3.27 ± 0.18	.14	NS	6.64 ± 0.37	5.42 ± 0.31	.03	S
KW% BW	1.56 ± 0.04	1.46 ± 0.06	.18	NS	1.91 ± 0.08	1.65 ± 0.07	.03	S
Renal cyst vol (ml)	0.39 ± 0.05	0.31 ± 0.09	.06	NS	0.72 ± 0.06	0.31 ± 0.04	.04	S
Liver weight (gr)	13.4 ± 0.54	11.3 ± 0.43	.01	S	16.80 ± 0.77	15.39 ± 0.81	.27	NS
LW% BW	5.59 ± 0.12	5.03 ± 0.11	.01	S	4.83 ± 0.10	4.69 ± 0.18	.58	NS
Renal fibrosis	1.69 ± 0.53	1.25 ± 0.42	.12	NS	2.00 ± 0.32	1.81 ± 0.46	.45	NS
Liver fibrosis	2.44 ± 0.18	2.08 ± 0.38	.035	S	2.30 ± 0.27	2.19 ± 0.26	.48	NS

14-week feeding protocol	*n* = 12	*n* = 10	*P*-value and significance	

Body weight (gr)	315 ± 8.7	323 ± 9.7	.58	NS
Kidney weight (gr)	4.57 ± 0.13	3.91 ± 0.16	.004	S
KW% BW	1.46 ± 0.06	1.21 ± 0.04	.004	S
Renal cyst vol (ml)	0.66 ± 0.06	0.42 ± 0.09	.035	S
Liver weight (gr)	20.2 ± 0.76	16.0 ± 0.05	.0002	S
LW% BW	6.47 ± 0.35	4.86 ± 0.11	.001	S
Renal cortical fibrosis	2.69 ± 0.10	2.33 ± 0.11	.026	S
Renal medullary fibrosis	2.17 ± 0.10	2.09 ± 0.10	.569	NS
Liver fibrosis	2.46 ± 0.07	2.58 ± 0.04	.171	NS

PCK rats were fed on control or pioglitazone-supplemented diet (20 mg/kg BW) from weeks 3–10 (7 weeks) or 4–18 (14 weeks). The values given are averages ± SEM. Abbreviations: KW% BW: total kidney weight as a percentage of total body weight; renal cyst vol: the estimated renal cystic volume; LW% BW: liver weight as a percentage of total body weight. Fibrosis was scaled on a 1–4 point scale using deidentified picrosirus red stained slides of transverse kidney sections as described in the methods section. *P*-values are for the comparison of control versus pioglitazone-supplemented diets by Students *t*-test. *P* less than  .05 is considered significant. NS: not significant.

**Table 2:** Effect of pioglitazone (4 mg/kg body weight) on fibrocystic disease in the PCK rat model.

7-week feeding protocol	Female animals				Male animals			
	Control *n* = 8	Pioglitazone *n* = 8	*P*-value and significance		Control *n* = 5	Pioglitazone *n* = 8	*P*-value and significance	
Body weight (gr)	239 ± 9.48	236 ± 7.89	.82	NS	347 ± 9.50	342 ± 6.31	.68	NS
Kidney weight (gr)	3.74 ± 0.22	3.38 ± 0.19	.23	NS	6.64 ± 0.37	5.53 ± 0.20	.014	S
KW% BW	1.56 ± 0.04	1.43 ± 0.06	.103	NS	1.91 ± 0.08	1.61 ± 0.05	.007	S
Renal cyst vol	0.39 ± 0.05	0.30 ± 0.03	.016	S	0.71 ± 0.06	0.50 ± 0.03	.006	S
Liver weight (gr)	13.4 ± 0.54	12.4 ± 0.59	.24	NS	16.80 ± 0.77	15.16 ± 0.49	.085	NS
LW% BW	5.59 ± 0.12	5.24 ± 0.11	.043	S	4.83 ± 0.10	4.42 ± 0.12	.040	S
Renal fibrosis	1.69 ± 0.53	1.50 ± 0.27	.38	NS	2.00 ± 0.35	1.38 ± 0.44	.022	S
Liver fibrosis	2.44 ± 0.18	2.31 ± 0.37	.39	NS	2.30 ± 0.27	2.36 ± 0.24	.68	NS

PCK rats were fed on control or pioglitazone-supplemented diet (4 mg/kg BW) from weeks 3–10 (7 weeks). The values given are averages ± SEM. Abbreviations: KW% BW: total kidney weight as a percentage of total body weight; renal cyst vol: the estimated renal cystic volume in ml; LW% BW: liver weight as a percentage of total body weight. Fibrosis was scaled on a 1–4 point scale using deidentified picrosirus red stained slides of transverse kidney sections as described in the methods section. *P*-values are for the comparison of control versus pioglitazone-supplemented diets by Students *t*-test. *P* less than  .05 is considered significant. NS: not significant.

**Table 3:** Serum profiles from PCK female mice—20 mg/kg BW Pioglitazone weeks 4–18.

	Normal range	Control diet *n* = 12	Pio diet *n* = 10	*P*-value and significance	
Na^+^ mM	141–148	140 ± 0.66	139 ± 0.76	.442	NS
K^+^ mM	4.3–5.9	4.92 ± 1.82	4.6 ± 0.53	.687	NS
Cl^−^ mM	101–108	99.7 ± 0.54	100.5 ± 0.72	.357	NS
Calcium mg/dl	9.6–11.2	10.3 ± 0.14	9.6 ± 0.36	.060	NS
BUN mg/dL	11–17	13.6 ± 0.53	12.4 ± 0.48	.118	NS
Creatinine mg/dL	0.4–0.7	0.33 ± 0.01	0.34 ± 0.03	.754	NS
Glucose mg/dL	120–186	162 ± 6.00	146.5 ± 4.59	.055	NS
Alkaline phos. U/L	65–117	315 ± 11.18	355 ± 26.67	.060	NS
Alanine aminotransferase U/L	25–45	62 ± 3.55	70.0 ± 6.71	.171	NS
Asparagine aminotransferase U/L	72–116	136 ± 12.98	139.5 ± 20.5	.302	NS
Bilirubin mg/dL	0.2–2	0.41 ± 0.06	0.32 ± 0.04	.281	NS
Total protein gr/dL	6.4–7.5	5.5 ± 0.11	5.6 ± 0.11	.486	NS
Albumin gr/dL	3.5–5.3	1.13 ± 0.04	1.39 ± 0.03	.0001	S

Values are given as means ± SEM. Control: PCK rats on the control diet; PIO: PCK rats on a diet containing 20 mg/kg body weight pioglitazone.

*n*: number of animals *P*-values greater than  .05 were considered nonsignificant (NS).
